# Geographic distribution of the *cagA*, *vacA*, *iceA*, *oipA* and *dupA* genes of *Helicobacter pylori* strains isolated in China

**DOI:** 10.1186/s13099-021-00434-4

**Published:** 2021-06-15

**Authors:** Zhijing Xue, Hong Yang, Dongxing Su, Xiangfeng Song, Xin Deng, Changhong Yu, Chunhua Sun, Lihua He, Yuanhai You, Yanan Gong, Dongjie Fan, Lu Sun, Xiurui Han, Ruyue Fan, Maojun Zhang, Xiaomei Yan, Jiaming Qian, Jianzhong Zhang

**Affiliations:** 1 State Key Laboratory of Infectious Disease Prevention and Control, Collaborative Innovation Center for Diagnosis and Treatment of Infectious Diseases, Chinese Center for Disease Control and Prevention, National Institute for Communicable Disease Control and Prevention, Beijing, China; 2 Peking Union Medical College Hospital, Peking Union Medical College, Chinese Academy of Medical Sciences, Beijing, China; 3 The Second Nanning People’s Hospital, Nanning, Guangxi Zhuang Autonomous Region, Nanning, China; 4 Department of Gastroenterology, Rushan People’s Hospital, Rushan, Shandong China; 5 Yiyang Central Hospital, Yiyang, Hunan China; 6 The First Affiliated Hospital of Jiamusi Medical University, Jiamusi, Heilongjiang China; 7 The People’s Hospital of Huzhu Tu Ethnic Autonomous County, Haidong, Qinghai China

**Keywords:** *Helicobacter pylori*, Genotype, Virulence genes, PCR, China

## Abstract

**Background:**

There are geographic variations in the genotypes of *Helicobacter pylori* (*H. pylori*) *cagA*, *vacA*, *iceA*, *oipA* and *dupA*. The aim of the study was to investigate the distribution of these genotypes among *H. pylori* strains from five regions of China and their association with clinical outcomes.

**Materials and methods:**

Gastric biopsy specimens were obtained from 348 patients with different gastrointestinal diseases in the five regions of China. The regional distribution was 89 patients from Shandong, 91 from Guangxi, 57 from Hunan, 58 from Qinghai and 53 from Heilongjiang. The presence of *cagA*, *vacA*, *iceA*, *oipA* and *dupA* genotypes was determined by polymerase chain reaction (PCR) from *H. pylori* DNA.

**Results:**

A total of 269 *H. pylori* isolates were obtained, of which 74 isolates were from Shandong, 78 from Guangxi, 46 from Hunan, 33 from Qinghai and 38 from Heilongjiang. The *cagA*-positive status was predominant in the five regions. The predominant *vacA* genotypes were s1c (73.4%), m2 (70.6%) and i1 (92.9%). In strains from Shandong, s1a and m1 were dominant. By contrast, s1c was dominant in Guangxi and i1 was dominant in Hunan and Heilongjiang. The prevalence of m2 subtype in Qinghai (78.8%) was significantly higher than that in other regions (P < 0.05). The predominant *iceA* genotype was *iceA1* and the frequency of *iceA1* was significantly more prevalent in Hunan than in other regions (P < 0.05). The *oipA* status “on” gene was more frequent in Shandong (91.9%) and Guangxi (91%) than in Heilongjiang (71.7%) (P < 0.05). Conversely, the *dupA*-positive status was less than half in Shandong (31.1%) and Guangxi (15.4%), whereas it was 73.9% in Hunan and 81.8% in Qinghai (P < 0.001). There were no significant associations between the *cagA*, *vacA*, *iceA*, *oipA* genotypes and clinical outcomes. The *dupA*-positive strains were more common in peptic ulcer disease (PUD) patients than in non-ulcer dyspepsia (NUD) patients in Shandong and Guangxi (P < 0.05), but the association was not observed in other geographic regions.

**Conclusions:**

There was significant geographic diversity of *H. pylori* genotypes in different regions of China and the presence of *dupA* gene can be considered as a marker for the development of gastroduodenal diseases. However, the *cagA*, *iceA*, *vacA* and *oipA* genes cannot be regarded for prediction of the clinical presentation of *H. pylori* infection in China.

**Supplementary Information:**

The online version contains supplementary material available at 10.1186/s13099-021-00434-4.

## Background

*Helicobacter pylori* (*H. pylori*) is a chronic infectious pathogen that can lead to gastroduodenal diseases such as chronic gastritis, peptic ulcer disease (PUD), gastric carcinoma (GC) and mucosa associated lymphoid tissue (MALT) lymphoma [41]. Owing to the carcinogenicity of *H. pylori*, it was classified as a grade I carcinogen by the World Health Organization [3]. It has been proved that more than half of the world’s population are infected with *H. pylori* and the prevalence of *H. pylori* has been declining in Western countries, whereas the prevalence has plateaued at a high level in developing countries [21]. *H. pylori* is characterized by genetic diversity, but the clinical symptoms caused by different strains are variable and considered to be related to the genetic susceptibility and living environment of the host, mainly due to the bacterial virulence factors [40].

Several *H. pylori* virulence factors, such as *cagA*, *vacA*, *iceA*, *oipA* and *dupA* have been identified to play an important role in the pathogenicity of *H. pylori* [24]. The CagA (cytotoxin-associated gene A) has been considered as an important carcinogen and *cagA*-positive strains can increase the risk of PUD or GC. There are EPIYA segments in the CagA C-terminal region, which are the tyrosine phosphorylation sites of CagA protein. According to the difference of the amino acid sequences flanking the EPIYA motifs, CagA C-terminal region can be divided into four different segments: EPIYA-A, EPIYA-B, EPIYA-C and EPIYA-D [20].

VacA (vacuolating cytotoxin A), which can induce vacuolation and multiple cellular activities, is encoded by *vacA* gene, which has distinct alleles [13]. Although *vacA* is present in all *H. pylori* strains, it shows allelic variation in three main regions: the signal (s) region (s1a, s1b and s1c, s2), the intermediate (i) region (i1 and i2) and the middle (m) region (m1 and m2) [37]. The different combination of s and m regions determines the production of cytotoxic activity and constitutes mosaic gene structure. Strains with the genotype s1m1 produce high levels of toxin in vitro, followed by s1m2, while s2m1 strains produce low toxicity and s2m2 strains produce little or no toxin [8]. It has been shown that s1m2 strains that contain the i1 allele are vacuolating, whereas strains that contain the i2 allele are non-vacuolating [17]. Studies have shown that s1m1 subtype is highly correlated with PUD and GC [19, 31, 38]. Additional studies also found strains containing the *vacA* i1 allele can increase the risk of GC [32]. There are geographic variations in the distribution of *vacA* genotypes in different regions. Many researches have shown that *vacA* s1a and s1c are predominant in Asia and northern Europe, whereas s1b is common in South America, Southern Europe and South Africa [14, 47]. These differences may lead to diversity in prevalence of gastroduodenal diseases in different geographic regions.

The *iceA* (induced by contact with epithelium) has two main allelic variants: *iceA1* and *iceA2*, which also has a particular geographic distribution [22]. The *iceA1* was common in Japan and Korea while the *iceA2* was predominant in the America, Colombia and Europe [35]. The OipA (outer inflammatory protein A) increases inflammatory response by affecting interleukin 8 (IL-8) production. The *oipA* functional status is regulated by slipped-strand mispairing that is based on the number of CT dinucleotide repeats in the signal sequences of the gene (switch “on” = functional and switch “off” = nonfunctional) [45]. Studies have shown that the prevalence of *oipA* in duodenal ulcer (DU) and GC is higher, suggesting that *oipA* is not only associated with inflammation, but also the development of GC [46]. The *dupA* (duodenal ulcer promoting gene A), first recognized as a marker of *H. pylori* specific disease, can induce DU and inhibit GC [2].

China is a country with large population, wide area and high incidence of *H. pylori* infection. In addition, the incidence rate of GC in China is higher than that in the Western countries. Heilongjiang and Qinghai provinces are high risk areas of GC, which are located in the northeast and northwest of China respectively, the mortality rate of GC ranges from 40 to 70 per 100,000 persons, compared to 10 to 20 per 100,000 persons in Guangxi and Hunan, low risk areas of GC, which are located in the South and Central South of China respectively [10]. Shandong province is located in the east of China and the crude mortality rate of GC was 49 per 100,000 persons, accounting for 21% of all malignant cancers [28]. At present, there are many studies focusing on the relationship between *H. pylori* virulence factors and clinical outcomes in China. However, only a few studies regarded information on the relationship between *H. pylori* virulence genotypes and different geographic regions. We therefore investigated the distribution of *vacA*, *cagA*, *iceA*, *oipA* and *dupA* genotypes in different regions of China and their association with clinical outcomes.

## Results

A total of 269 *H. pylori* isolates out of 348 gastric biopsy specimens from five geographic regions of China were obtained, of which 74 isolates were from Shandong, 78 from Guangxi, 46 from Hunan, 33 from Qinghai and 38 from Heilongjiang. At endoscopy 21 patients presented with PUD. The remaining 248 patients were diagnosed as having non-ulcer dyspepsia (NUD). No patients with GC were included in the study. *H. pylori* virulence genes *cagA*, *vacA*, *iceA*, *oipA* and *dupA* were detected by polymerase chain reaction (PCR) in all isolates and *H. pylori* genotypes results were summarized in Table 1.

**Table 1:** Distribution of virulence genotypes among 269 *H. pylori*-positive patients with different geographic regions

Genetypes	No. of isolates					
	SD (n = 74)	GX (n = 78)	HN (n = 46)	QH (n = 33)	HL (n = 38)	Total (n = 269)
*cagA*	70(94.6)	74(94.9)	46(100)	33(100)	38(100)	261(97)
*vacA*						
s1	72(97.3)	78(100)	45(97.8)	30(90.9)	38(100)	263(97.8)
s2	2(2.7)	0	1(2.2)	3(9.1)	0	6(2.2)
m1	**30(40.5)**	14(17.9)	10(21.7)	6(18.2)	12(31.6)	72(26.8)
m2	**43(58.1)**	61(78.2)	35(76.1)	26(78.8)	25(65.8)	190(70.6)
i1	66(89.2)	71(91)	46(100)	**29(87.9)**	38(100)	250(92.9)
i2	8(10.8)	7(9)	0	4(12.1)	0	19(7.1)
s1i1m1	22(29.7)	12(15.4)	10(21.7)	6(18.2)	12(31.6)	62(23)
s1i1m2	42(56.8)	53(67.9)	34(73.9)	22(66.7)	25(65.8)	176(65.4)
s1i2m1	7(9.6)	2(2.6)	0	0	0	9(3.3)
s1i2m2	0	5(6.4)	0	1(3)	0	6(2.2)
s2i1m1	0	0	0	0	0	0
s2i1m2	1(1.4)	0	1(2.2)	0	0	2(0.7)
s2i2m1	1(1.4)	0	0	0	0	1(0.4)
s2i2m2	0	0	0	3(9.1)	0	3(1.1)
*iceA1*	49(66.2)	50(64.1)	**38(82.6)**	24(72.7)	26(68.4)	187(69.5)
*iceA2*	12(16.2)	20(25.6)	8(17.4)	6(18.2)	8(21.1)	54(20.1)
*oipA*"on"	68(91.9)	71(91)	41(89.1)	30(90.9)	**27(71.1)**	237(88.1)
*oipA*"off"	3(4.1)	2(2.6)	0	0	0	5(1.9)
*dupA*	23(31.1)	**12(15.4)**	34(73.9)	27(81.8)	25(65.8)	121(45)

Isolates were from five regions of China: Shandong (SD), Guangxi (GX), Hunan (HN), Qinghai (QH) and Heilongjiang (HL)

Values in parentheses are percentages

Numbers in boldface type indicate a significant difference compared with other regions

### *cagA* genotypes

**Fig. 1:**
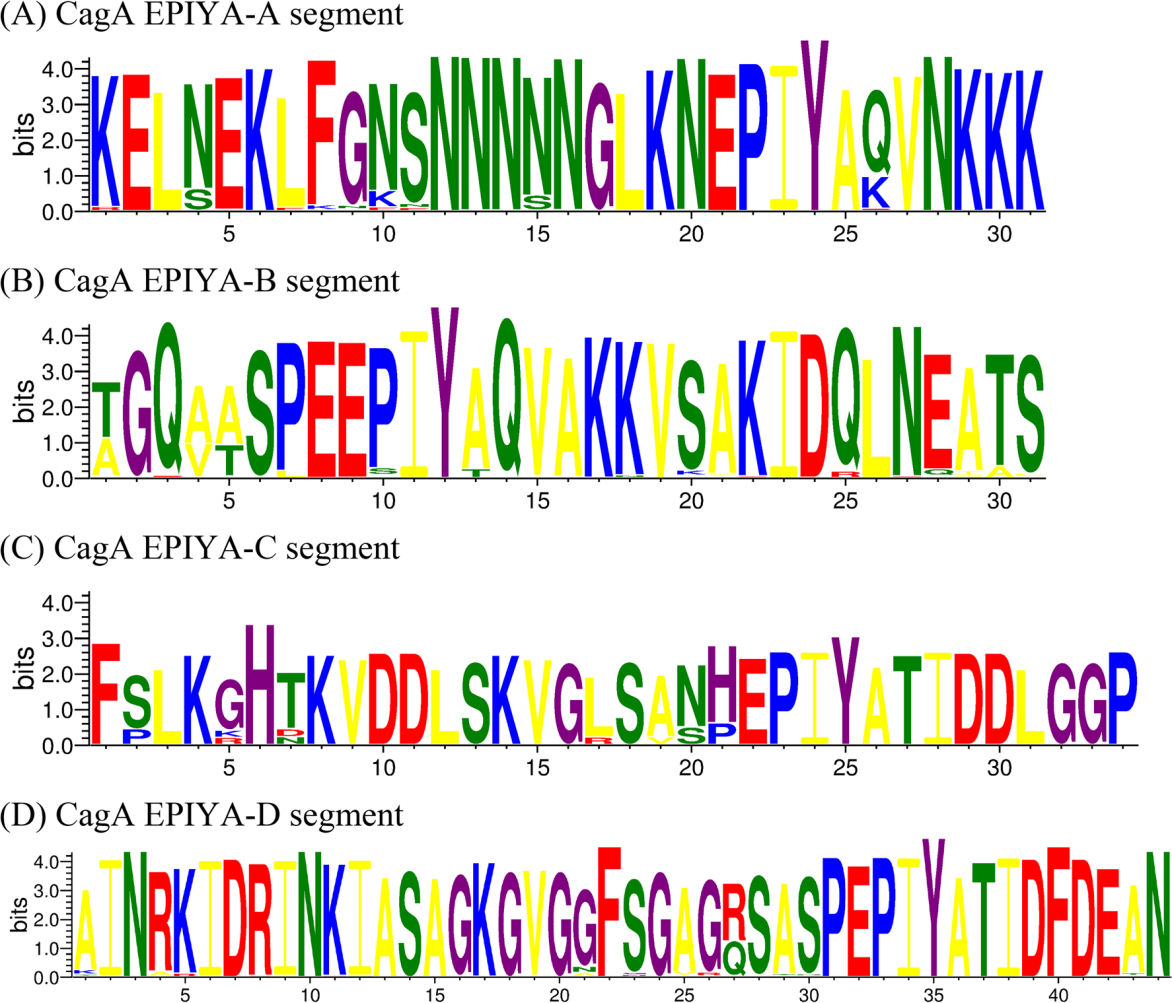
EPIYA segment types in East Asian-type and Western-type CagA from Chinese strains

Overall, 261(97%) patients were infected with *cagA*-positive strains (Table 1). Of these *cagA*-positive strains, 70(94.6%) strains were isolated from Shandong, 74 (94.9%) from Guangxi, 100% from Hunan, Qinghai and Heilongjiang. 96.6% (252/261) of the *cagA* genes detected were East Asian type, whereas only 9 strains were Western type. 55.6% (5/9) Western strains were from Heilongjiang and there was a significant correlation between the type ABC and Heilongjiang isolates (χ^2^ = 15.512, P < 0.01). The distribution of CagA sequence types was shown in Table 2. There were 2–4 EPIYA motifs in CagA C-terminus, and 94.3% [(238 + 8)/261] of the strain sequences had three EPIYA segments. There were obvious differences between segment EPIYA-C and EPIYA-D when analyzed using the WebLogo 3 (Fig. 1).

**Table 2:** Distribution of CagA sequence types in different geographic regions of China

Regions	CagA sequence types					Total
	AB	ABD	AABD	ABC	ABCC	
SD	9	59	1	1	0	70
GX	0	71	1	1	1	74
HN	0	46	0	0	0	46
QH	0	32	0	1	0	33
HL	3	30	0	**5**	0	38
Total	12(4.6)	238(91.2)	2(0.8)	8(3)	1(0.4)	261

Isolates were from five regions of China: Shandong (SD), Guangxi (GX), Hunan (HN), Qinghai (QH) and Heilongjiang (HL)

Values in parentheses are percentages

Number in boldface type indicates a significant difference compared with other regions

The phylogenetic tree was constructed from *cagA* 3’ variable region sequences. As shown in the Additional file 1, the phylogenetic tree diverged into two lineages. In the lineage one, *H. pylori* 26695 was clustered with 9 Western type strains from different regions. In the second lineage, *H. pylori* GZ27 was clustered with East Asian strains from five regions. The phylogenetic analysis did not reveal any association between a particular disease and a specific *cagA* sequence. The *cagA* gene was present in 97.2% and 95.2% of *H. pylori* strains isolated from patients with NUD and PUD, respectively (Table 3). There was no statistical difference between the *cagA* genotypes and clinical outcomes irrespective of the different geographic regions (χ^2^ = 0.669, P > 0.05).

**Table 3:** Frequency of 269 *H. pylori* virulence genotypes in patients with NUD and PUD

Genetypes	No. of isolates											
	SD (n = 74)		GX (n = 78)		HN (n = 46)		QH (n = 33)		HL (n = 38)		Total (n = 269)	
	NUD (61)	PUD (13)	NUD (75)	PUD (3)	NUD (42)	PUD (4)	NUD (32)	PUD (1)	NUD (38)	PUD (0)	NUD (248)	PUD (21)
*cagA*	58(95.1)	12(92.3)	71(94.7)	3(100)	42(100)	4(100)	32(100)	1(100)	38(100)	0	241(97.2)	20(95.2)
*vacA*												
s1	60(98.4)	12(92.3)	75(100)	3(100)	41(97.6)	4(100)	30(93.8)	0	38(100)	0	244(98.4)	19(90.5)
s2	1(1.6)	1(7.7)	0	0	1(2.4)	0	2(6.6)	1(100)	0	0	4(1.6)	2(9.5)
m1	26(42.6)	4(30.8)	13(17.3)	1(33.3)	10(23.8)	0	6(18.8)	0	12(31.6)	0	67(27)	5(23.8)
m2	34(55.7)	9(69.2)	59(78.7)	2(66.7)	31(73.8)	4(100)	25(78.1)	1(100)	25(65.8)	0	174(70.2)	16(76.2)
i1	56(91.8)	10(76.9)	68(90.7)	3(100)	42(100)	4(100)	29(90.6)	0	38(100)	0	233(94)	17(81)
i2	5(8.2)	3(23.1)	7(9.3)	0	0	0	3(9.4)	1(100)	0	0	15(6)	4(19)
s1i1m1	21(34.4)	1(7.7)	11(14.7)	1(33.3)	10(23.8)	0	6(18.8)	0	12(31.6)	0	60(24.2)	2(9.5)
s1i1m2	33(54.1)	9(69.2)	51(68)	2(66.7)	30(71.4)	4(100)	22(68.8)	0	25(65.8)	0	161(64.9)	15(71.4)
s1i2m1	5(8.2)	2(15.4)	2(2.7)	0	0	0	0	0	0	0	7(2.8)	2(9.5)
s1i2m2	0	0	5(6.7)	0	0	0	1(3.1)	0	0	0	6(2.4)	0
s2i1m1	0	0	0	0	0	0	0	0	0	0	0	0
s2i1m2	1(1.6)	0	0	0	1(2.4)	0	0	0	0	0	2(0.8)	0
s2i2m1	0	1(7.7)	0	0	0	0	0	0	0	0	0	1(4.8)
s2i2m2	0	0	0	0	0	0	3(9.4)	0	0	0	0	0
*iceA1*	40(65.6)	9(69.2)	48(64)	2(66.7)	35(83.3)	3(75)	23(69.7)	1(100)	26(68.4)	0	172(69.4)	15(71.4)
*iceA2*	9(14.8)	3(23.1)	19(25.3)	1(33.3)	7(16.7)	1(25)	6(18.2)	0	8(21.1)	0	49(19.8)	5(23.8)
*oipA*"on"	55(90.2)	13(100)	68(90.7)	3(100)	37(88.1)	4(100)	29(87.9)	1(100)	27(71.1)	0	216(87.1)	21(100)
*oipA*"off"	3(4.9)	0	2(2.7)	0	0	0	0	0	0	0	5(2)	0
*dupA*	**15(24.6)**	**8(61.5)**	**10(13.3)**	**2(66.7)**	32(76.2)	2(50)	26(78.8)	1(100)	25(65.8)	0	108(43.5)	13(61.9)

Isolates were from five regions of China: Shandong (SD), Guangxi (GX), Hunan (HN), Qinghai (QH) and Heilongjiang (HL)

Values in parentheses are percentages

Numbers in boldface type indicate a significant difference compared clinical outcomes (NUD and PUD) in each geographic region group with respect to *H. pylori* genotype

### *vacA* subtypes

**Fig. 2:**
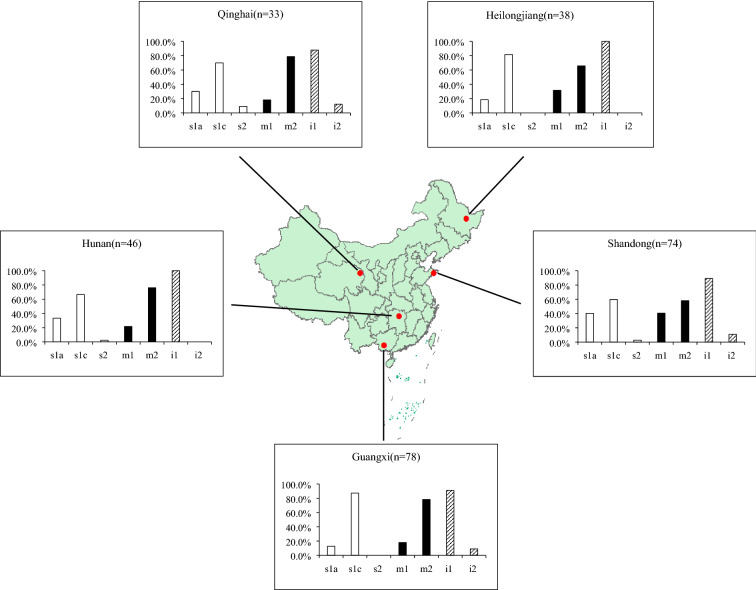
Distribution of *vacA* s, m and i region genotypes of *H. pylori* strains from different regions of China. The prevalence of each type (s1a, s1c, s2, m1, m2, i1 and i2) is given as a percentage of the total number of strains (shown in parentheses)

The most common *vacA* s region genotype was s1 (97.8%): 97.3% of strains from Shandong, 97.8% from Hunan, 90.9% from Qinghai, 100% from Guangxi and Heilongjiang. Regardless of geographic origins and clinical outcomes, most of the *vacA* s1 strains were of the s1c subtype (73.4%), while the s1a subtype was detected in 70 (26.6%) and the s1b subtype was not detected in our study (Table 1; Fig. 2). The prevalence of s1a in the isolates from Shandong was 40.3%, significantly higher (χ^2^ = 15.930, P < 0.01) than that in Guangxi (12.8%), Hunan (33.3%), Qinghai (30%) and Heilongjiang (18.4%). Conversely, the s1c subtype was significantly (χ^2^ = 19.806, P < 0.01) more frequent in isolates from Guangxi (87.2%) than the other four regions. For the *vacA* m region, 190 patients (70.6%) were infected with m2 strains and 72 (26.8%) were infected with m1 strains. The frequency of the m2 subtype in Qinghai (78.8%) was significantly higher (χ^2^ = 9.900, P < 0.05) than in Shandong (58.1%). In contrast, the frequency of m1 subtype in Shandong (40.5%) was significantly higher (χ^2^ = 12.539, P < 0.05) than the other four regions. All the *H. pylori*-infected patients were successfully detected for the *vacA* i region, in which 250 (92.9%) patients were infected with i1 strains, while the remaining 19 (7.1%) patients were infected with i2 strains (Table 1). The prevalence of i1 allele was significantly higher (χ^2^ = 9.687, P < 0.05) in Hunan and Heilongjiang than in other regions. We also examined the different combinations of *vacA* s, m and i alleles in patients. The dominant *vacA* subtype combination was s1i1m2 (65.4%) in the five regions, but no statistical significance was noted (χ^2^ = 4.168, P > 0.05). There were also no statistical differences between the *vacA* subtypes and clinical outcomes (Table 3).

## *iceA* status

Overall, *iceA1* was detected in 187 (69.5%) of all 269 isolates examined and *iceA2* was found in 54 isolates (20.1%) (Table 1). The *iceA1* frequency was significantly more prevalent in Hunan (82.6%) than in the other four regions (χ^2^ = 11.358, P < 0.05). The *iceA2* was present in 25.6% and 21.1% of *H. pylori* strains isolated from Guangxi and Heilongjiang, respectively, whereas only 16.2% of isolates from Shandong were infected with *iceA2*-positive strains. However, the difference was not statistically significant (χ^2^ = 3.204, P > 0.05). There was also no association between the *iceA* status and clinical outcomes in the five regions of China (Table 3).

### *oipA* status

242 (90%) isolates were positive with *oipA* set primers, and, overall, 88.1% had a functional status “on” (Table 1). A total of 10 *oipA* CT repeat patterns were identified (Table 4). The pattern containing (3 + 1) CT repeats was the most frequently associated with the “on” status (125/237, 52.7%), and the pattern with 5 CT repeats was the most prevalent for a nonfunctional (“off”) *oipA* gene (3/5, 60%). The *oipA* functional status “on” was more prevalent in Shandong isolates than in Heilongjiang isolates (χ^2^ = 8.060, P < 0.05). Overall, 87.1% of NUD patients and 100% of PUD patients were infected with *oipA* functional status “on” strains, the difference was not statistically significant (χ^2^ = 3.561, P > 0.05) (Table 3). When the analyses were carried out in each geographic region, the differences were also not statistically significant.

**Table 4:** Frequency of the *oipA* CT repeat patterns

Sequence of signal peptide encoding region of *oipA*	No. of CT	No. of isolates
**“On” status**		
ATGAAAAAAGCTCTCTTACTAACTCTCTTTTTCTCGTTTTGGCTCCACGCTGAA	3 + 1	125
M K K A L L L T L F F S F W L H A E		
ATGAAAAAAACCCTTTTACTCTTTCTGTCTTTCTCGTTTTGGCTCCACGCTGAA	2 + 1 + 1	82
M K K T L L L F L S F S F W L H A E		
ATGAAAAAAGCTCTCTTACTAACTCTCTTTCTCTCGTTTTGGCTCCACGCTGAA	3 + 2	14
M K K A L L L T L F L S F W L H A E		
ATGAAAAAAACCCTTTTACTCACTCTTTCTCTCTCGTTTTGGCTCCACGCTGAA	2 + 3	1
M K K T L L L T L S L S F W L H A E		
ATGAAAAAAACCCTTTTACTCTTTCTGTCTCTCTCGTTTTGGCTCCACGCTGAA	1 + 3	5
M K K T L L L F L S L S F W L H A E		
ATGAAAAAAGCTCTCTTACTAATTCTCTTTTTCTCGTTTTGGCTCCACGCTGAA	2 + 1	1
M K K A L L L I L F F S F W L H A E		
ATGAAAAAAGCTCTCTTACTAACTCTCTCTCTCTCGTTCTGGCTCCACGCTGAA	6	9
M K K A L L L T L S L S F W L H A E		
**“Off” status**		
ATGAAAAAAGCTCTCTTACTAACTCTCTCTCTCGTTTTGGCTCCACGCTGA	5	3
M K K A L L L T L S L V L A P R *		
ATGAAAAAAGCTCTCTTACTCTCTCTCTCTCTCGTTCTGGCTCCATGCTGA	7	1
M K K A L L L S L S L V L A P C *		
ATGAAAAAAGCTCTCTTACTAACTCTCTCTCTCTCTCTCGTTTTGGCTCCACGCTGA	8	1
M K K A L L L T L S L S L V L A P R*		

The underline represents the sequences of *oipA* CT repeat patterns

⁎Indicates stop codon

### *dupA* status

121 (45%) patients were infected with *dupA*-positive strains (Table 1). The *dupA*-positive strains were present in 73.9% of Hunan, 81.8% of Qinghai and 65.8% of Heilongjiang. In contrast, only 31.1% of Shandong and 15.4% of Guangxi strains were infected with *dupA*-positive *H. pylori* (χ^2^ = 72.497, P < 0.001). Overall, 43.5% of patients with NUD and 61.9% of those with PUD were infected with *dupA*-positive strains (Table 3). The *dupA*-positive strains were significantly more common in PUD patients than in NUD patients in Shandong (χ^2^ = 6.830, P < 0.01) and Guangxi (χ^2^ = 4.254, P < 0.05). In contrast, the *dupA*-positive strains were more common in NUD patients (76.2%) than in PUD patients (50%) in Hunan, but the difference was not statistically significant (χ^2^ = 1.299, P > 0.05).

## Discussion

### Geographic distribution versus genotypes

The present study investigated the *cagA*, *vacA*, *iceA*, *oipA* and *dupA* genotypes of *H. pylori* isolated from patients living in different geographic regions of China. Our study demonstrated that there was obvious geographic diversity of *H. pylori* genotypes in China, emphasizing that even within a country genetic diversity still existed. There are at least two reasons for the difference in *H. pylori* strains among different geographic regions. One is the accumulation of mutations in *H. pylori* strains in different regions, and the other is that there may be adaptive evolutionary selection between *H. pylori* strains and their hosts.

In China, more than 90% of *H. pylori* strains carry *cagA* gene, which may be the reason why the incidence rate of GC in China is higher than that in Western countries. In the present study, 97% patients were infected with *cagA*-positive strains. This result was similar to studies in other Asian countries and some regions of China where the prevalence of *cagA*-positive strains was above 90% ([34, 48]. However, this was different from reports in some European and American countries where the prevalence of *cagA*-positive strains ranged from 50 to 70% [15]. Furthermore, we found that the majority of CagA types were East Asian type, only 3.4% were Western type. Some studies have shown that Western type CagA was the most frequent type in Mongolian and Russia patients and all *H. pylori* strains from GC patients possessed Western type CagA [36, 42]. In our study, 55.6% (5/9) of the Western CagAs were from Heilongjiang, which may be due to human migration or direct transmission.

In the present study, there was a high prevalence of s1c 73.4% in the *vacA*-positive strains, similar to previous study in other regions of China [43]. However, the result was slightly different from some reports in which the prevalence of s1c was a little lower [14, 43]. The *vacA* s1b was not detected in our study, whereas the prevalence of s1b subtype was almost 100% in South America, 80% in Spain and Portugal strains, very few in East Asia [14]. The *vacA* s2 was prevalent in Africa and consistent with studies in some European and American countries [5, 14], but the s2 detected in this study was very low, further revealing the geographic diversity of *vacA* gene. The presence of *vacA* m1 strains was significantly higher in Shandong, which may be the reason for the high incidence of gastric cancer. These findings were different from some countries such as Japan, Korean, Singapore and some European and American strains [14, 47, 50], suggesting the differences between Chinese and foreign strains. In the *vacA* i region, the i1 subtype was dominant in the five regions and this result was consistent with those of studies that include patients from some countries, such as Japan and South Korea, where the prevalence of i1 subtype was over 95% [12, 26].

The prevalence of *iceA1* was 69.5% in our study, consistent with studies reported from Thailand and Korea [11, 27]. The *oipA* status was regulated by strip strand repairing based on the number of CT nucleotide repeats in the signal sequences. The present study revealed a high prevalence of strains with *oipA* status “on” genes (88.1%) regardless of the geographic origins and clinical outcomes, which was similar to previous studies [49].The presence of *dupA* gene was different in distinct geographic regions, such as 84.8% in the South Africa, 43.7% in the Belgium and 70% in the United States [4]. Similarly, in the present study, the prevalence of the *dupA* was also different in distinct geographic regions of China. We detected 81.8% *dupA*-positive isolates in Qinghai, while the lower prevalence of *dupA* (15.4%) was in Guangxi and 31.1% in Shandong. The reasons for the difference in prevalence of *dupA* gene in the five geographic regions of China are unclear.

### Clinical outcome versus genotypes

The present study did not reveal any associations between the *cagA*, *vacA*, *iceA* and *oipA* genotypes and clinical outcomes. These results were consistent with other reports from China [43, 44], but was different from many studies in Western countries [7]. One important reason for the difference might be due to large genomic difference of the *H. pylori*. Some researchers considered that the *iceA1* was more common in patients with PUD while the *iceA2* was most frequently isolated from NUD patients [18, 33]. However, our study showed that the presence of the *iceA* gene was not associated with clinical outcomes. OipA is an important outer membrane protein that is closely related to severe inflammatory response and the induction of IL-8 secretion. Studies showed that the *oipA* status “on” was expressed in most strains isolated from patients with PUD, suggesting that it could be helpful in predicting the clinical presentation of *H. pylori* infection in different regions [46]. In this study, the presence of *oipA* status “on” had no correlation with clinical outcomes. Other outer membrane proteins, such as BabA, SabA and HomB, widely exist in different strains, which might play a vital role in the pathogenesis of *H. pylori* [1]. These virulence factors need further study.

Surprisingly, the *dupA*-positive strains were significantly more common in PUD patients than in NUD patients in Shandong and Guangxi (P < 0.05). The results were different from previous studies in which there was no association between the presence of *dupA* and clinical diseases [39]. Conversely, the prevalence of the *dupA*-positive strains was more common in NUD patients (76.2%) than in PUD patients (50%) in Hunan, but the difference was not statistically significant. Therefore, further molecular epidemiology researches in other populations will help to study the association between *dupA* gene and clinical outcomes.

## Conclusions

The present study investigated the distribution of *H. pylori* virulence genotypes in five regions of China and their association with clinical outcomes. There was a reverse correlation between the *dupA* gene and PUD. However, we could not reveal clear associations of the *cagA*, *iceA*, *oipA* and *vacA* genotypes with clinical outcomes in any of the studied regions.

## Materials and methods

### Study subjects

A total of 348 patients were involved in this study including 89 patients from Rushan People’s Hospital (Weihai, Shandong Province, China), 91 from the Second Nanning People’s Hospital (Nanning, Guangxi Province, China), 57 from Yiyang Central Hospital (Yiyang, Hunan Province, China), 58 from the People’s Hospital of Huzhu Tu Ethnic Autonomous County (Haidong, Qinghai Province, China) and 53 from The First Affiliated Hospital of Jiamusi University (Jiamusi, Heilongjiang Province, China). Their gastric biopsy specimens were obtained during upper gastrointestinal endoscopy with informed consent. This study was approved by Ethical Committee of National Institute for Communicable Disease Control and Prevention Chinese Center for Disease Control and Prevention (approval No. ICDC-2013001).

### *H. pylori* culture and DNA extraction

Gastric biopsy specimens were homogenized thoroughly in brain heart infusion (BHI) broth and then streaked onto the Karmali blood agar base plates under a biological safety cabinet (Thermo Scientific). The Karmali Agar base (Oxoid, CM 0935) was supplemented with 5% defibrinated sheep blood and 1% combined antibiotics comprising of trimethoprim (150 mg/L), vancomycin (125 mg/L), amphotericin B (100 mg/L) and polymyxin B (100 mg/L). The plates were incubated at 37 °C under microaerobic conditions (5% O_2_, 10% CO_2_ and 85% N_2_) for 3–5 days. *H. pylori* colonies were identified according to its morphological characteristics, negative Gram staining and positive for catalase, oxidase, and urease. The identified *H. pylori* was subcultured to single colonies and then preserved in sterile BHI broth with 20% glycerol and frozen at − 80 °C until the genomic DNA was extracted with the QIAamp DNA Mini Kit (Qiagen, Germany) according to the manufacturer’s instructions. The extracted DNA was stored at − 20℃ and used directly for PCR.

### PCR amplification

The PCR reaction was carried out in a total volume of 25 μL containing forward and reverse primers (0.2 μM each), 2 ng/μL DNA template, 12.5 μL Go Taq® Green Master Mix (Promega, USA) and 9.5 μL nuclease-free water. The amplification was as follows: initial denaturation at 94 °C for 5 min and then denaturation at 94 °C for 30 s, primer annealing at 54, 56, 60, 56 and 62 °C for *cagA*, *iceA*, *dupA*, *vacA* (s1/s2, s1a, s1b, s1c, m1, m2 and i1, i2) and *oipA*, respectively, for 30 s and extension at 72 °C for 40 s. All reactions were performed through 35 cycles. The final cycle included a final extension at 72 °C for 10 min. The presence of the *cagA*, *iceA* and *dupA* genes was determined by PCR as previously described [9, 23, 30]. The genotypes of *vacA* s1/s2, s1a, s1b, s1c, m1, m2 and i1, i2 were also determined by PCR as previously described [6, 16, 29, 37]. The *oipA* gene was detected by PCR, which was additionally sequenced in order to define its functional status as either “on” or “off”. The signal sequences of *oipA* gene including the CT repeats were amplified by using primer pairs as described previously [25]. The primers used to amplify the targeted genes were summarized in Table 5. The amplified products were analyzed in 1% agarose gel containing 1 × TAE, stained with GelStain and visualized by electrophoresis at 110 V for 30 min using the gel documentation system (Bio-Rad, USA).

**Table 5:** Primers used for PCR amplification of *cagA*, *vacA*, *iceA*, *oipA* and *dupA* genes

Gene	Primer	Primer sequence (5'–3')	Product size (bp)
*cagA*	*cagA*3'-F	TGCGTGTGTGGCTGTTAGTAG	593–752
	*cagA*3'-R	CCTAGTCGGTAATGGGTTGT	
*vacA*			
s1/s2	Vs-F	ATGGAAATACAACAAACACAC	259/286
	VA-R	CTGCTTGAATGCGCCAAAC	
s1a	Vs1a-F	GTCAGCATCACACCGCAAC	190
	VA-R	CTGCTTGAATGCGCCAAAC	
s1b	Vs1b-F	AGCGCCATACCGCAAGAG	187
	VA-R	CTGCTTGAATGCGCCAAAC	
s1c	Vs1c-F	CTCTCGCTTTAGTGGGGYT	213
	VA-R	CTGCTTGAATGCGCCAAAC	
m1	Vm1-F	GGCCCCAATGCAGTCATGGAT	240
	Vm1-R	GCTGTTAGTGCCTAAAGAAGCAT	
m2	Vm2-F	GGAGCCCCAGGAAACATTG	352
	Vm2-R	CATAACTAGCGCCTTGCAC	
i1	Vi-F	GTTGGGATTGGGGGAATGCCG	495
	Vi1-R	TTAATTTAACGCTGTTTGAAG	
i2	Vi-F	GTTGGGATTGGGGGAATGCCG	495
	Vi2-R	GATCAACGCTCTGATTTGA	
*iceA1*	*iceA1*-F	GTGTTTTTAACCAAAGTATC	246
	*iceA1*-R	CTATAGCCAGTCTCTTTGCA	
*iceA2*	*iceA2*-F	GTTGGGTATATCACAATTTAT	229
	*iceA2*-R	TTGCCCTATTTTCTAGTAGGT	
*oipA*	*oipA*-F	CGCGGAAAGGAACGGGTTTT	519
	*oipA*-R	TTAGCGTCTAGCGTTCTGCC	
*dupA*	*dupA*-F	GACGATTGAGCGATGGGAATAT	971
	*dupA*-R	CTGAGAAGCCTTATTATCTTGTTGG	

### Sequencing and bioinformatics analysis

Positive PCR products were sent to the Beijing Genomics Institute (BGI) for purification and sequencing. The nucleotide sequences of the *cagA* 3’ variable region and *oipA* were submitted to China National Microbiological Data Center. The accession numbers are NMDCN0000M60 to NMDCN0000ME4 and NMDCN0000ME5 to NMDCN0000MLM, respectively. DNA sequences were edited by EditPlus version 5.3.0 and the edited nucleotide sequences were subjected to translation using BioEdit version 7.2.5. The EPIYA segment types of CagA were analyzed using the program WebLogo 3 (http://weblogo.threeplusone.com/). Neighbor-Joining phylogenetic tree was constructed from *cagA* 3’ variable region nucleotide sequences using MEGA version 7.0.18 and bootstrap analysis was performed with 1000 replications. The Western strain 26695 (GenBank No. CP003904) and the East Asian strain GZ27 (GenBank No. KR154756) were used as reference sequences.

### Statistical analysis

Statistical data were analyzed by SPSS software version 20. The chi-square test and Fisher’s exact test were used to assess the relationship between specific genotype and geographic origins and clinical outcomes. P-value < 0.05 was considered of a statistically significant difference.

## Supplementary Information

**Additional file 1:** The phylogenetic tree of *cagA* gene.

## Data Availability

The Western strain 26695 and East Asian strain GZ27 were downloaded from NCBI (https://www.ncbi.nlm.nih.gov/).

## Abbreviations

*H. pylori*: *Helicobacter pylori*
PCR: Polymerase chain reaction
GC: Gastric carcinoma
PUD: Peptic ulcer disease
NUD: Non-ulcer dyspepsia
MALT: Mucosal-associated lymphoid tissue
*cagA*: Cytotoxin-associated gene A
*vacA*: Vacuolating cytotoxin A
*iceA*: Induced by contact with epithelium
*oipA*: Outer inflammatory protein A
*dupA*: Duodenal ulcer promoting gene A
